# Inhibitor of caspase-activated DNase expression enhances caspase-activated DNase expression and inhibits oxidative stress-induced chromosome breaks at the mixed lineage leukaemia gene in nasopharyngeal carcinoma cells

**DOI:** 10.1186/s12935-015-0205-1

**Published:** 2015-05-24

**Authors:** Siaw Shi Boon, Sai-Peng Sim

**Affiliations:** Faculty of Medicine and Health Sciences, Universiti Malaysia Sarawak, Kota Samarahan, Sarawak Malaysia

**Keywords:** CAD, ICAD, Oxidative stress, Nasopharyngeal carcinoma, *MLL*, Chromosome breaks

## Abstract

**Background:**

Nasopharyngeal carcinoma (NPC) is commonly found in Asia, especially among the Chinese ethnic group. Chromosome rearrangements are common among NPC patients. Although the mechanism underlying the chromosome rearrangements in NPC is unclear, various mechanisms including activation of caspase-activated DNase (CAD) were proposed to contribute to chromosome rearrangements in leukaemia. Activation of CAD can be initiated by multiple agents, including oxidative stress, which is well implicated in carcinogenesis. CAD is the main enzyme that causes DNA fragmentation during apoptosis, and CAD is also implicated in promoting cell differentiation. In view of the role of oxidative stress in carcinogenesis and CAD activation, and since CAD was suggested to contribute to chromosome rearrangement in leukaemia, we hypothesise that oxidative stress-induced CAD activation could be one of the mechanisms that leads to chromosome rearrangements in NPC.

**Methods:**

SUNEI cells were treated with various concentrations of H_2_O_2_ for different period of time to ensure that cells undergo H_2_O_2_-induced *MLL* gene cleavage. Transfections with hCAD, mCAD, mutant hCAD, or cotransfection with hCAD and mICAD, and cotransfection with mutant hCAD and mICAD were performed. Gene expression was confirmed by Western blotting and *MLL* gene cleavage was assessed by inverse polymerase chain reaction (IPCR).

**Results:**

Treatment with H_2_O_2_ clearly induces cleavages within the *MLL* gene which locates at 11q23, a common deletion site in NPC. In order to investigate the role of CAD, CAD was overexpressed in SUNE1 cells, but that did not result in significant changes in H_2_O_2_-induced *MLL* gene cleavage. This could be because CAD requires ICAD for proper folding. Indeed, by overexpressing ICAD alone or co-expressing ICAD with CAD, Western blotting showed that CAD was expressed. In addition, ICAD overexpression also suppressed H_2_O_2_-induced *MLL* gene cleavage, suggesting a possible role of CAD in initiating chromosome cleavage during oxidative stress.

**Conclusions:**

Oxidative stress mediated by H_2_O_2_ induces cleavage of the *MLL* gene, most likely via the caspase-activated DNase, CAD, and CAD expression requires ICAD. Since the *MLL* gene is located at 11q23, a common deletion site in NPC, thus stress-induced CAD activation may represent one of the mechanisms leading to chromosome rearrangement in NPC.

## Background

Nasopharyngeal Carcinoma (NPC) is one of the most common cancers in Asia especially in South East Asia, particularly among the Chinese ethnic group [1, 2]. It is well associated with Epstein-Barr virus infection, environmental, dietary, genetic and epigenetic factors [3–7]. The epigenetic effect was demonstrated by promoter methylation of tumour suppressor gene, which was suggested to play a role in the growth and invasion of NPC cells [8]. Furthermore, various chromosome rearrangements are found in NPC, including chromosome gains and losses [9]. A number of studies have identified potential tumour suppressor genes and oncogenes [10]. Nevertheless, the mechanism (s) leading to the non-random chromosome rearrangements in NPC is still elusive. Various mechanisms of chromosome rearrangements have been proposed in other malignancies. These include mechanisms involving *Alu* repeats [11], V (D) J recombination [12], DNA topoisomerase II [13] and apoptosis [14, 15]. More recently, caspase-activated DNase (CAD) is also implicated in chromosome rearrangement in NPC [16].

Apoptosis is a naturally occurring cell death process that is important in various biological systems [17]. Apoptosis is characterised by a series of distinct morphological and biochemical changes [18]. Most of these morphological changes result from the activity of a class of cysteine proteases, called caspases [19]. A number of caspases have been identified, where about two-thirds of them function in apoptosis [20]. Caspases normally exist as inactive pro-enzymes. When apoptois is triggered, caspases are converted into active enzyme to cleave a subset of proteins [21], either inactivating or activating the target proteins [19]. During apoptosis, while the caspases are activated, the genomic DNA is fragmented into high-molecular-weight (HMW) DNA as well as the smaller fragments known as the internucleosomal DNA ladder [22]. These HMW DNA fragments of 50–300 kb correspond to the DNA-loop structures [23], which interact with the nuclear matrix via the matrix-attachment region or scaffold-associated region (MAR/SAR) sequence [24]. Thus, the HMW DNA formation during apoptosis appears to be DNA loop excision at the MAR/SAR sequence at the base of DNA loop [25]. One of the key enzymes in apoptosis is caspase-activated DNAase (CAD) [26]. Normally CAD exists as an inactive complex with its inhibitor, the Inhibitor of CAD (ICAD). During apoptosis induction, ICAD is cleaved by caspase-3, thus releasing the activated CAD, allowing it to cleave the genomic DNA into HMW DNA as well as internucleosomal DNA ladder [27]. CAD seems to be playing multiple roles. On one hand it is the apoptotic nuclease, one the other hand, it was also found to play a role in chromosome rearrangement commonly found in leukaemia [14, 15, 28]. In addition, CAD was also shown to promote cell differentiation by inducing DNA strand breaks [29].

Apoptotic DNA fragmentation can be induced by a range of stimuli including cytotoxic drugs [15], Fas ligands [30], virus infection [31] and oxidative stress [32]. Oxidative stress induces apoptotic DNA fragmentation in endothelial cells under an ATP rich environment [33] as well as in skeletal muscle myoblasts [34]. It was also found to induce the formation of High Molecular Weight (HMW) DNA fragmentation in leukaemic cells [35]. Oxidative stress produces reactive oxygen species (eg. H_2_O_2_, hydroxyl radical and superoxide), that cause injuries on various cellular macromolecules. These damages have been proposed to contribute to the development of cancer [36, 37]. Oxidatively damaged DNA are repaired by the Base Excision Repair pathway (BER) [38] which involves the function of *hOGG1 and XRCC1* [39, 40]. Polymorphism of these two genes was shown to be associated with elevated risk of NPC [41], supporting the role of oxidative stress in NPC development.

Knowing that oxidative stress is contributing to NPC, which contains multiple chromosome rearrangements; while oxidative stress also induces apoptotic DNA fragmentation and the apoptotic nuclease, CAD, has been implicated in chromosome rearrangements; thus, we hypothesise that, oxidative stress-induced CAD activation may result in chromosome rearrangement which partly contributes to NPC development. In general, chromosome rearrangement requires chromosome breaks. Since CAD is the major enzyme in apoptotic chromosome fragmentation, we intend to investigate the effect of CAD expression on hydrogen peroxide-induced chromosome breaks within the mixed lineage leukaemia (*MLL*) gene. The *MLL* gene is chosen based on the following criteria: (1) it is located at 11q23 [42], a commonly deleted site in NPC [9]; (2) it is commonly translocated in leukaemia [43] and (3) expression of the Epstein-Barr virus latent membrane protein 1 (*LMP1*) gene induces cleavage of the *MLL* gene [16].

In this report, we show that oxidative stress induced cleavage of the *MLL* gene. Expression of CAD alone did not enhance *MLL* gene cleavage. Interestingly, expression of ICAD not only enhanced CAD expression, but also inhibited *MLL* gene cleavage, supporting the role of CAD in *MLL* gene cleavage during oxidative stress.

## Results

### Hydrogen peroxide (H_2_O_2_) induces cleavage of the *MLL* gene

**Fig. 1:**
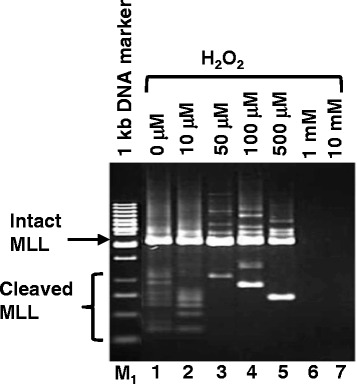
Hydrogen peroxide (H_2_O_2_) induces *MLL* gene cleavage in NPC cells. SUNE1 cells were treated with either 0 μM, 10 μM, 50 μM, 100 μM, 500 μM, 1 mM or 10 mM of H_2_O_2_ for 20 h (lanes 1–7 respectively). Genomic DNA was extracted and modified for IPCR as described in Materials and Methods. DNA was digested with *Msc* I during the modification. Side arrow shows the 2.2 kb band resulting from amplification of the intact *MLL* gene. Side bracket shows bands smaller than 2.2 kb resulting from amplification of cleaved *MLL* gene. M_1_ represents 1 kb DNA marker

In order to examine the contribution of H_2_O_2_-induced chromosome breaks leading to chromosome rearrangement in NPC, SUNE1 cells were treated with various concentrations of H_2_O_2_ for 20 h. Genomic DNA was modified and analysed by nested inverse polymerase chain reaction (IPCR). Based on the primer position, an intact *MLL* would yield a 2.2 kb band while any band smaller than 2.2 kb represents amplification of the cleaved *MLL*. As shown in Fig. 1, cells treated with 50 μM, 100 μM and 500 μM of H_2_O_2_ resulted in cleavage of the *MLL* gene as indicated by the presence of distinct bands smaller than 2.2 kb (Lanes 3–5 respectively). Treatment with 1 and 10 mM (Lanes 6 and 7) results in no amplification at all, this is probably due to extensive damage of the template DNA at high H_2_O_2_ concentration.

### Transient transfection with normal and mutant CAD did not enhance H_2_O_2_-induced *MLL* gene cleavage

**Fig. 2:**
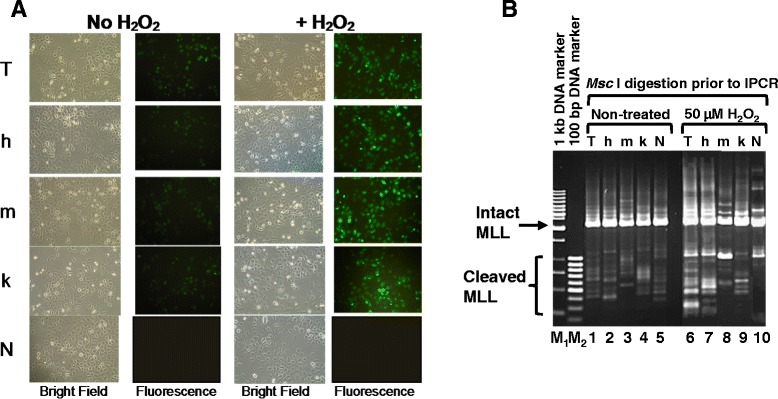
Transient transfection with normal and mutant CAD do not enhance H_2_O_2_-induced *MLL* gene cleavage. **a** Microscopic morphology of transiently transfected SUNE1 cells. SUNE1 cells were transfected with either the vector pTracer, T; pTracer-hCAD, h; pTracer-mCAD, m; or pTracer-hCAD (k157Q), k. A mock transfection (no DNA), N, was included as control. Transfected cells were either treated with 50 μM of H_2_O_2_ for 8 h at 17 h post-transfection, or left untreated. Observation was then carried out with light microscopy and fluorescence microscopy. **b** Nested IPCR detection of *MLL* gene cleavage. SUNE1 cells were transfected with either the vector pTracer, T (Lanes1 and 6), pTracer-hCAD, h (Lanes 2 and 7), pTracer-mCAD, m (Lanes 3 and 8) or pTracer-hCAD (k157Q), k (Lanes 4 and 9). A mock transfection (no DNA), N (Lanes 5 and 10) was included as control. The cells were either non-treated (Lanes 1–5) or treated with 50 μM of H_2_O_2_ for 8 h at 17 h post-transfection. Genomic DNA extracted was modified for IPCR as described in Materials and Methods. Side arrow shows the 2.2 kb band resulting from amplification of the intact *MLL* gene. Side bracket shows bands smaller than 2.2 kb resulting from amplification of cleaved *MLL* gene. M_1_ represents 1 kb DNA marker. M_2_ represents 100 bp DNA marker

Since H_2_O_2_ clearly induces cleavage of the *MLL* gene, we would like to investigate if this cleavage is mediated by CAD. Overexpression of CAD in HeLa cells was shown to result in nucleosomal DNA ladder formation [44], thus we chose to transiently transfect both HeLa and and SUNE1 cells, follow by H_2_O_2_ treatment, to examine if CAD expression enhances H_2_O_2_-induced *MLL* gene cleavage. As shown in Fig. 2a, the various transfections were successful, with expression of the green fluorescence protein (GFP) clearly shown for each transfection, in the presence or absence of H_2_O_2_ Nested IPCR was then performed to detect *MLL* gene cleavage in transfected cells. To our surprise, comparing with transfection with vector alone (Fig. 2b, lane 6), neither transfection with human CAD nor mouse CAD enhances H_2_O_2_ -induced *MLL* gene cleavage (Fig. 2b, lanes 7 and 8). Similarly, transfection with the mutant human CAD (Fig. 2b, lanes 9) did not show significant difference from transfection with wild-type human or mouse CAD (Fig. 2b, lanes 7 and 8). In the absence of H_2_O_2_, the various transfection results in similar background cleavage (Fig. 2b, lanes 1–5). The same transfections were done in HeLa cells and similar results were observed (data not shown).

### ICAD expression enhances CAD expression

**Fig. 3:**
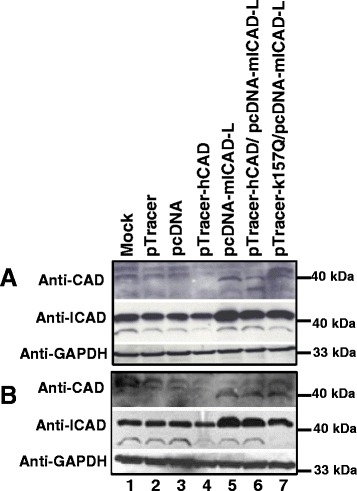
ICAD expression enhances CAD expression. Two sets of SUNE1 cells were transiently transfected with vector pTracer (Lane 2), pcDNA (Lane 3), pTracer-hCAD (Lane 4), pcDNA-mICAD-L (Lane 5), or cotransfection with pTracer-hCAD/pcDNA-mICAD-L (Lane 6) and pTracer-hCAD (K157Q)/pcDNA-mICAD-L (Lane 7). A mock transfection (No DNA) was included as control (Lane 1). Transfected cells were either not treated (Panel **a**) or treated with 50 μM of H_2_O_2_ for 6 h at 17 h post-transfection (Panel **b**). Protein was extracted as detailed in Materials and Methods. Expression of CAD, ICAD and GAPDH was each analysed by anti-CAD, anti-ICAD and anti-GAPDH respectively

Although CAD overexpression in HeLa was shown to enhance DNA nucleosomal ladder formation [44], ICAD was also suggested to be the chaperon for CAD [26]. In view of this, co-transfection of CAD and ICAD was carried out in SUNE1 cells. As shown in Fig. 3, human CAD transfection alone in deed did not show enhanced CAD expression (Panel A, lane 4). However, transfection with full-length mouse ICAD alone greatly enhanced CAD expression (Panel A, lane 5). At the same time, co-transfection wtih human CAD and mouse ICAD, or co-transfection with mutant human CAD and mouse ICAD also enhanced CAD expression (Panel A, lanes 6 and 7). Western blot with anti-ICAD clearly shows that ICAD expression was successful (Panel A, lanes 5–7). Expression of GAPDH shows consistent loading in all samples (Panel A, lanes 1–7). To ensure that subsequent H_2_O_2_ treatment does not change the expression pattern, transfected cells were also treated with H_2_O_2_ at 17 h post-trasfection. The results show that the expression pattern for the different transfection is similar to that in the absence of H_2_O_2_ (Panel B, lanes 1–7).

### ICAD expression inhibits *MLL* gene cleavage

**Fig. 4:**
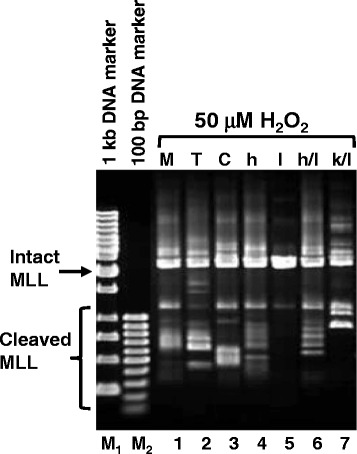
ICAD expression inhibited H_2_O_2_-induced *MLL* gene cleavage. SUNE1 cells were transiently transfected with vector pTracer (Lane 2), pcDNA (Lane 3), pTracer-hCAD (Lane 4), pcDNA-mICAD-L (Lane 5), or cotransfection with pTracer-hCAD/pcDNA-mICAD-L (Lane 6) and pTracer-hCAD (K157Q)/pcDNA-mICAD-L (Lane 7). A mock transfection (No DNA) was included as control (Lane 1). Transfected cells were treated with 50 μM of H_2_O_2_ for 6 h at 17 h post-transfection. Genomic DNA was extracted and processed for IPCR as detailed in Materials and Methods. Side arrow shows the 2.2 kb band resulting from amplification of the intact *MLL* gene. Side bracket shows bands smaller than 2.2 kb resulting from amplification of cleaved *MLL* gene. M_1_ represents 1 kb DNA marker. M_2_ represents 100 bp DNA marker

Since ICAD expression enhances CAD expression, we intend to investigate this effect on *MLL* gene cleavage in the presence of H_2_O_2_. There are two possibilities, one is ICAD expression enhances CAD expression (as shown in previous section), and thus enhances *MLL* gene cleavage. Alternatively, due to high level of ICAD overexpressed, it may also inhibit the activity of CAD though CAD’s expression was enhanced. Indeed, ICAD overexpression inhibits cleavage of the *MLL* gene in the presence of 50 μM H_2_O_2_ (Fig. 4, lane 5). Co-transfection with ICAD and human CAD or co-transfection with ICAD and mutant human CAD do not show obvious inhibition of *MLL* gene cleavage (Fig. 4, lanes 6 and 7 respectively). This could be due to lesser amount of ICAD DNA being used during co-transfection as compared to ICAD transfection alone. Lesser amount of ICAD DNA is required during co-transfection to avoid DNA toxicity during transfection. At the same time, this may also be due to more CAD expressed from the transfected plasmid in addition to endogenous CAD.

## Discussion

The apoptotic nuclease, caspase activated DNase (CAD) was suggested to play a direct role in mediating chromosome translocation in leukaemia [15, 28]. Although chromosome rearrangements are commonly found in nasoparyngeal carcinoma (NPC), the mechanism leading to these rearrangements is yet unclear. Our earlier data showed that expression of Epstein-Bar virus (EBV) latent membrane protein 1 (LMP1) induced cleavage of the mixed lineage leukemia (*MLL*) gene at 11q23, which was significantly reduced by caspase inhibitor [16]. Since 11q23 is a commonly deleted site in NPC, thus it was suggested that virus-induced CAD activation could be involved in the initiation of chromosome rearrangement in NPC. Oxidative stress was found to be a contributing factor to NPC [41] and is known to induce apoptotic DNA fragmentation [32], thus, the current study extended to investigate the role of CAD in oxidative stress-induced chromosome breaks within the *MLL* gene in NPC cells.

Although oxidative stress was suggested to be a contributing factor to NPC, it was unclear of the molecular mechanism. In this current study, we clearly demonstrated that hydrogen peroxide induced chromosome breaks within the *MLL* gene. Since *MLL* locates at 11q23, a common deletion site in NPC [9], thus our result suggests that oxidative stress could be initiating chromosome aberration by inducing chromosome breaks at the *MLL* gene. *MLL* gene contains MAR/SAR sequences [45] which has been implicated in chromosome rearrangement [46], while oxidative stress has also been shown to poison DNA topoisomerase II, resulting in high molecular weight (HMW) DNA fragmentation [35]. High molecular weight (HMW) DNA fragmentation appears to result from excision of the chromosomal DNA loops [25], whose base is interacting with the nuclear matrix via the MAR/SAR sequence [47]. Although it was suggested that, the loop excision was mediated by DNA topoisomerase II [35], it is also known that DNA topoisomerase II interacts with CAD at the nuclear matrix [48], thus not excluding the role of CAD in the event. This is supported by our result that hydrogen peroxide induces cleavage of the *MLL* gene near the MAR/SAR region.

Overexpression of CAD in HeLa cells was shown to induce nucleosomal DNA ladder formation [44], thus we chose to express CAD in HeLa and SUNE1 cells and then assess for H_2_O_2_-induced chromosome breaks. No obvious difference was observed among the different transfectants as detailed in the result section. This could be due to the reason that ICAD is acting as a chaperone for the proper folding of CAD during its expression [26]. This result seems to contradict with the observation of Mukae *et al*. [44]. However, Mukae *et al*. [44] employed both the anti-Fas antibody and actinomycin D and successfully demonstrated that overexpression of CAD alone in HeLa results in nucleosomal DNA ladder formation [44]. Anti-Fas antibody activates the death receptor pathway during apoptosis [49], while actinomycin D targets DNA topoisomerase II, which activates the mitochondrial pathway during apoptosis [50]. In the current study, H_2_O_2_ is the sole apoptosis inducer, while H_2_O_2_-induced apoptosis is CD-95 independent [51] and it induces apoptosis through the mitochondrial pathway [52]. This subtle difference may explain the different observations in these two studies.

During protein synthesis in a cell-free system, CAD was recovered as a complex with ICAD, suggesting that ICAD acts as a chaperon for CAD [26]. This is further supported by our result that, expression of ICAD enhanced the expression of endogenous and exogenous CAD. In the current study, mouse ICAD and human CAD were used. Thus it seems that the chaperone activity of mouse ICAD can be applied to human CAD. This is supported by the fact that mouse ICAD and human ICAD are almost identical at the N-terminal domain [44], the so called CAD domain [53]. This domain is required for the chaperon activity of ICAD, as well as for CAD/ICAD association during formation of the heterotetrameric structure of (CAD/ICAD) _2_ [54]. This kind of chaperone-assisted protein expression is also evidenced in microorganism such as *Escherichia coli* [55].

Our result shows that ICAD expression enhances CAD expression, while we also hypothesise that CAD is mediating the H_2_O_2_-induced *MLL* gene cleavage. Thus, in order to investigate the final effect of ICAD expression in terms of *MLL* gene cleavage, the transfected cells were treated with H_2_O_2_ and assessed by IPCR. Our result clearly shows that although ICAD expression induced endogenous CAD expression, it has also extensively reduced H_2_O_2_-induced *MLL* gene cleavage. This is obviously explained by the fact that ICAD expression was much higher than CAD as shown by the Western blot result. Thus, the endogenous CAD expressed was inhibited by the overexpressed exogenous CAD. Similarly, ICAD expression also inhibited Fas ligand-induced and staurosporine-induced DNA fragmentation [27]. This supports our hypothesis that CAD could be involved in *MLL* gene cleavage during oxidative stress. This is also consistent with CAD’s role in chromosome translocation in treatment-related leukemia [28]. In the absence of apoptotic stimuli, CAD is in complex with ICAD and thus stays inactive [26]. Both CAD and ICAD share a homologous N-terminal domain of about 80 amino acids [44]. This domain is known as the CAD domain. The CAD domain of CAD and ICAD interact with each other during CAD/ICAD heterodimeric complex formation, and this interaction is important for the proper folding of CAD [53, 56]. Upon induction of apoptosis by apoptotic stimuli, the caspase cascade is activated, with caspase 3 cleaving ICAD, thus releasing the activated CAD [27]. CAD/ICAD complex is normally mobile in the nucleosol of dividing cells, but during apoptosis induction, CAD becomes progressively immobilised and associates with the nuclear matrix [57]. Nuclear matrix is the anchorage site for the organisation of DNA-loop structure in the cell [47]. The base of DNA loops contain DNA sequences known as the matrix-attachment region or scaffold-associated region (MAR/SAR) and it is through these sequences that DNA is interacting with the nuclear matrix [24]. With CAD being associated with the nuclear matrix during apoptosis [57], it is obviously in close proximity to the MAR/SAR sequence of DNA and potentially cleave the DNA when it is activated. The region of DNA involved in this study falls within the MAR/SAR sequence, thus supporting the role of CAD in mediating *MLL* gene cleavage during oxidative stress.

## Conclusions

From the current findings, we concluded that oxidative stress mediated by H_2_O_2_ induces cleavage of the *MLL* gene, most likely via the activity of CAD, and CAD expression requires ICAD. Since the *MLL* gene is located at 11q23, a common deletion site in NPC, thus stress-induced CAD activation may represent one of the mechanisms leading to chromosome rearrangement in NPC.

## Methods

### Cell lines and materials

HeLa cell line was a gift from Dr. Edmund Sim Ui Hang of Universiti Malaysia Sarawak. Nasopharyngeal carcinoma cell line, SUNE1 was generously provided by Professor Dr. Sam Choon Kook previously from Universiti Malaya. Plasmid clones for hCAD, mCAD and mICAD were generous gifts from Professor Dr. Shigekazu Nagata of Osaka Bioscience Institute. DNA Polymerase I, Large (Klenow) fragment, T4 DNA Ligase and all the restriction enzymes were purchased from New England Biolabs (NEB, England). PCR primers were obtained from First Base Laboratories. Phusion DNA Polymerase was purchased from Finnzymes, Finland. Blood and Cell Culture DNA Mini-Prep kit and QIAquick Gel Extraction Kit were purchased from QIAGEN, Germany. All cell culture and trasfection reagents were obtained from GIBCO, Invitrogen. Protease inhibitor cocktail was purchased from Sigma, USA.

### Cell culture and H_2_O_2_ treatment

HeLa cells were maintained in Dulbecco’s Modified Eagle Medium (DMEM), which contains low glucose (1X), 1 g/L D-glucose, L-glutamine (2 mM) and 100 mg/L sodium pyruvate, supplemented with 10 % heat-inactivated fetal bovine serum (FBS), penicillin (100 U/ml) and streptomycin (100 μg/ml). Meanwhile, SUNE1cells were maintained in RPMI 1640 medium supplemented with 10 % heat-inactivated fetal bovine serum (FBS), L-glutamine (2 mM), penicillin (100 U/ml) and streptomycin (100 μg/ml). All cells were grown at 37 °C with 5 % CO_2_. HeLa and SUNE1 cells in 60 mm dishes were treated with 0, 10, 50, 100, 500, 1000 and 10,000 μM of H_2_O_2_ for 20 h. Cells were collected, genomic DNA was extracted using Blood and Cell Culture mini kit.

### Nested Inverse polymerase chain reaction (IPCR) detection of H_2_O_2_-induced chromosome breaks within the *MLL* gene

**Fig. 5:**
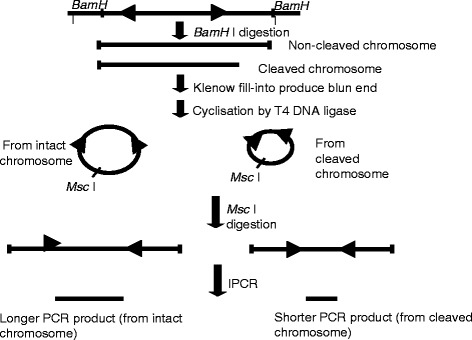
Flow chart showing DNA modification and IPCR. The arrow heads indicate the forward and reverse primers that were designed in opposite direction. *Bam*H I digestion yielded a mixture of intact chromosome and cleaved chromosome. Klenow fill-in produced blunt ended chromosome fragments which were then cyclilsed by T4 DNA ligase. The intact chromosome will become a large circle while the cleaved chromosome will become a smaller circle. Upon cyclisation, the primers are now in correct orientation for amplification. *Msc* I digestion cleaved both circles outside the amplification region, thus merely linearise the molecule. Amplification from intact *MLL* gene will produce longer PCR products while amplification from cleaved *MLL* gene will yield shorter PCR products

Figure 5 shows a summary of DNA modification as well as the IPCR. Briefly, extracted genomic DNA was digested with *Bam*H I (NEB, USA) at 37 °C overnight, followed by Klenow fill-in with DNA Polymerase I Large Fragment. Cyclisation by T4 DNA Ligase was performed and subsequently linearisation by *Msc* I. Nested IPCR was carried out with 150 ng of *Msc* I-digested template DNA, 50 pmol each of the primers, 200 μM each of the dNTP, 0.4 unit of Phusion polymerase and 1X of Phusion HF buffer. PCR cycle condition was: 1 cycle at 98 °C for 30 s, followed by 30 cycles at 98 °C for 10 s, 59 °C for 30 s, 72 °C for 15 s and a final cycle at 72 °C for 10 min. Second round PCR was performed with 2 μl of 5 time-diluted first round PCR products. PCR cycle condition was similar to the first round, except that the annealing and extension steps were carried out at 54 °C for 30 s and 72 °C for 11 s respectively. PCR products were analysed on 1 % agarose gel in 0.5X TBE buffer. The primers used were 5′-GAGAATCGCTTGAACCCAACAG-3′ and 5′-CTTGTGGGTCAGCAATTCCTTC-3′ in the first round, 5′-CCACTCCTTTATATTCCCATAGC-3′ and 5′-TCCTCCACTCACCTGATTC-3′ in the second round. Intact *MLL* gene will give rise to an amplification product of 2.2 kb while breaks within the region encompassed by the primers will produce amplification products smaller than 2.2 kb.

### Site-directed mutagenesis of human CAD (hCAD)

Site-directed mutagenesis of hCAD was performed using megaprimer site-directed mutagenesis protocol [58]. During the first round of PCR, pTracer-hCAD which carries the wild type human CAD gene was used as the template. Forward primer used was 5′-CGATTTCA**GAG**CCAGTCTGG-3′, which carries a mutation from Lysine (K), AAG to Glutamine (Q), GAG as highlighted in bold, while the reverse primer used was 5′-CG**TCTAGA**CTGGCGTTTCCG-3′ which carries an *Xba* I restriction site (in bold). An amplification product of 589 bp was produced and was used as the reverse primer (a megaprimer) in second round of PCR. The forward primer used in second PCR was 5′-CT**GGTACC**ATGCTCCAGAAG-3′ which carries a *Kpn* I restriction site (in bold). The final amplification product was a 1 kb fragment of mutated hCAD (K157Q) with *Xba* I and *Kpn* I restriction sites at the 5′ and 3′ ends respectively. Gel-purified amplification product was subsequently subcloned into pTracer producing pTracer-hCAD (K157Q). The subcloned gene was then confirmed by DNA sequencing.

### Transient transfection and co-transfection of NPC cells

SUNE1 cells were transfected with 2 μg each of the following: pTracer-hCAD carrying the wild type human CAD gene; pcDNA-mICAD-L carrying the full length wild type mouse ICAD; pTracer and pcDNA as controls. SUNE1 cells were also co-transfected with the following: pTracer-hCAD and pcDNA-mICAD-L; pTracer-hCAD (k157Q) carrying the mutant human CAD and pcDNA-mICAD-L in a ratio of 2:1 (1.33 μg: 0.67 μg). Mock transfection was also carried out where cells underwent transfection process in the absence of DNA. Transfection was performed with LipofectAMINE™ reagent and PLUS reagent (Invitrogen, Carlsbad, USA) following the manufacturer’s protocol.

### SDS PAGE and Western analysis

Attached cells were lysed with 0.5 ml of triple detergent mix (50 mM Tris.Cl, pH8; 150 mM sodium chloride; 0.02 % sodium azide; 0.1 % SDS; 1 % Nonidet P-40 and 0.5 % sodium deoxycholate) containing freshly added protease inhibitor cocktail and PMSF. Lysis was carried out on ice for 5 min, and then transferred to 1.5 ml microcentrifuge tubes. The lysate was further lysed by freezing in liquid nitrogen for 5 min followed by thawing at 37 °C. The lysate was vortexed vigorously for 1 min and the freez-thaw process was repeated for another 4 times. Centrifugation at 20,000x g was carried out for 10 min. Protein concentration in the supernatant was estimated using Bradford Assay. Fifty migrogram of each crude extract was boiled for 10 min in the presence of 2x SDS sample buffer (1X Tris.Cl, pH 6.8, 20 % glycerol, 4 % SDS, 0.2 % 2-merchaptoethanol, 0.001 % bromophenol blue). Samples were centrifuged, and analysed on 10 % SDS-polyacrylamide gel, followed by transfer onto polyvinylidene fluoride (PVDF) membrane (Millipore, Burlington, MA). Immunoblotting was performed with rabbit anti-CAD (Stressgen) at 1:1000 dilution, rabbit anti-ICAD (Stressgen) at 1:4000 dilution and rabbit anti-GAPDH (Santa Cruz, USA) at 1:4000 dilution. Goat anti-rabbit IgG HRP (Santa Cruz, USA) was used as the secondary antibody at 1:10,000 dilution. Chemiluminescent detection was carried out using the WestPico Chemiluminescent Substrate Kit (Pierce, USA) following the manufacturer’s protocol.
